# Morphology and molecules support the new monotypic genus *Parainvolucrella* (Rubiaceae) from Asia

**DOI:** 10.3897/phytokeys.180.67624

**Published:** 2021-08-03

**Authors:** Yi-Da Xu, Ming-Deng Yuan, Rui-Jiang Wang

**Affiliations:** 1 Key Laboratory of Plant Resources Conservation and Sustainable Utilization, South China Botanical Garden, Chinese Academy of Sciences, Guangzhou, Guangdong 510650, China South China Botanical Garden, Chinese Academy of Sciences Guangzhou China; 2 University of Chinese Academy of Sciences, Beijing 100049, China University of Chinese Academy of Sciences Beijing China

**Keywords:** new combination, palynology, *
Parainvolucrella
*, *
Scleromitrion
*, taxonomy

## Abstract

*Parainvolucrella* R.J. Wang, a new monotypic genus for *P.scabra* (Wall. ex Kurz) M.D.Yuan & R.J.Wang, new combination, is segregated from the *Hedyotis*-*Oldenlandia* complex, based on morphological and molecular evidence. Phylogenetically, the new genus is sister to *Scleromitrion*, from which it differs by a combination of morphological characters: herbaceous habit, terminal inflorescence with subtended leaves, heterostylous flowers, indehiscent fruits and pollen with double microreticulate tectum. A key to the genera of the *Hedyotis-Oldenlandia* complex in China is provided for further identification.

## Introduction

As one of the largest species groups of the family Rubiaceae, the *Hedyotis*-*Oldenlandia* complex contains hundreds of species distributed in the tropical and subtropical region worldwide. Due to morphological intermediacy and homoplasy, systematic studies in herbaceous Rubiaceae are very difficult (Gibbons 2020). The generic delimitation within this complex is complicated and controversial (Neupane et al. 2015) and historically disputed. The commonly shared morphological characters, such as four petals and calyx lobes, 2-celled ovaries with numerous ovules on axile placenta and capsular fruits made some studies treat this complex as one genus, *Hedyotis* L., in a broad sense (Lamarck 1792; Fosberg and Sachet 1991; Dutta and Deb 2004; Chen and Taylor 2011). Whereas, morphological differences in habit, inflorescence position, homo- or heterostylous flowers, dehiscent or indehiscent fruits, as well as the shape and ornamentation of seeds and pollen, provide unquestionable evidence to separate this complex into several small genera (Bremekamp 1952; Terrell et al. 1986; Terrell and Robinson 2003). Recent phylogenetic analyses, based on multiple nuclear and chloroplast DNA markers, revealed that this complex was polyphyletic and supported its subdivision into small genera (Groeninckx et al. 2009; Neupane et al. 2009; Guo et al. 2013; Wikström et al. 2013; Neupane et al. 2015; Gibbons 2020). Then the *Hedyotis* species in China fall into the following genera of *Debia* Neupane & N.Wikstr., *Dimetia* (Wight & Arn.) Meisn., *Edrastima* Raf., *Hedyotis*, *Involucrella* (Benth. & Hook.f.) Neupane & N.Wikstr., *Leptopetalum* Hook. & Arn., *Oldenlandia* L. and *Scleromitrion* (Wight & Arn.) Meisn. (Neupane et al. 2015; Wang 2018).

During our field investigation in Guangxi Zhuang Autonomous Region, we came across the species *Hedyotisscabra* Wall. ex Kurz, not recorded previously in China (Wei 2018), in bamboo forest nearby the Nonggang National Nature Reserve. This species has arbitrarily been treated as *Scleromitrionscabrum* (Wall. ex Kurz) Neupane & N.Wikstr. with insufficient morphological and molecular evidence (Neupane et al. 2015). Morphologically, it is similar to *Involucrellacoronaria* (Kurz) Neupane & N.Wikstr. for its terminal inflorescence subtended by four involucral leaves. Our subsequent morphological comparison and phylogenetic analysis, based on multiple DNA markers, support that this species represents a new genus.

## Materials and methods

Morphological characters of *Hedyotisscabra* were scored from living materials and dried specimens. All vouchers which we collected were deposited at the herbarium of South China Botanical Garden, Chinese Academy of Sciences (**IBSC**). Pollen and seeds were observed using scanning electron microscopy (JSM-6360LV) under 15.00 kV accelerating voltage. Pollen terminology for description followed Punt et al. (2007).

Methods of DNA extraction and PCRs followed Guo et al. (2011). Sequences of all taxa were downloaded from GenBank for molecular phylogenetic analysis, except for the newly added *Hedyotishainanensis*, *H.ovata*, and three samples of *Hedyotisscabra* (Table 1). Geneious v.11.0.3 (Kearse et al. 2012) was used for sequence alignment and MrModeltest 2.0 was applied for selecting the best-fit nucleotide substitution model (GTR+G+I) on the basis of the AIC criterion (Nylander 2004). Bayesian Inference (BI) was performed using MrBayes v.3.2.7 (Ronquist et al. 2012), with a calculation of posterior probabilities (PP) to each clade. The bootstrap (BS) values were obtained by IQ-TREE v. 2.0 (Nguyen et al. 2015) for Maximum Likelihood analyses based on the best-fit nucleotide substitution model (GTR+F+R3) selected by ModelFinder (Kalyaanamoorthy et al. 2017).

**Table 1.** Taxa, vouchers, localities and GenBank accession numbers of ITS, *pet*D, *rps*16, *trn*H-*psb*A and *trn*L-F sequences for phylogenetic analysis.

Taxon	Voucher (herbarium)	ITS	*pet*D	*rps*16	*trn*H-*psb*A	*trn*L-F
*Debiaovatifolia* (Cav.) Neupane & N. Wikstr.	China: Xing Guo & Ping Yang 20-1 (IBSC)	JF699940	JF700090	JX111309	JF699795	JX111382
*Dentellarepens* J.R. Forst. & G. Forst.	Australia: Andersson 2262 (GB)	AM939440	EU557693	AF333370	/	EU543091
*Dimetiaampliflora* (Hance) Neupane & N. Wikstr.	China: Ruijiang Wang et al. 1147 (IBSC)	JX111198	JX111086	JX111242	JX111161	JX111317
*Dimetiaauricularia* (L.) R.J. Wang	China: Ruijiang Wang & Yiding Gao 1185 (IBSC)	JF699904	JF700053	JX111298	JF699765	JX111372
Dimetia capitellata (Wall. ex G. Don) Neupane & N. Wikstr. var. capitellata	China: Xiangxu Huang et al. GBOWS1278 (IBSC)	JX111201	JX111089	JX111250	JX111164	JX111327
*Dimetiascandens* (Roxb.) R.J. Wang	China: Guo Xing & Ping Yang 10 (IBSC)	JF699949	JF700099	/	JF699804	/
*Edrastimatrinervia* (Retz.) Neupane & N. Wikstr.	Sri Lanka: F. Fagerlind 4338 (S)	HE657769	HE657652	HE649907	/	/
*Hedyotisacutangula* Champ. ex Benth.	China: Ruijiang Wang HA-02 (IBSC)	JX111197	JX111085	JX111241	JX111160	JX111316
*Hedyotiscantoniensis* F.C. How ex W.C. Ko	China: Ruijiang Wang et al. 1250 (IBSC)	JF976484	JF700061	JX111247	JF699773	JX111322
*Hedyotiscaudatifolia* Merr. & F.P. Metcalf	China: Ruijiang Wang et al. 1269 (IBSC)	JF699916	JF700065	JX111256	JF699777	JX111329
*Hedyotiseffusa* Hance	China: Ruijiang Wang et al. 1268_1 (IBSC)	JF699933	JF700083	JX111262	JF699790	JX111335
*Hedyotishainanensis* (Chun) W.C. Ko	China: Guobing Jiang & Xinxin Zhou 1121 (IBSC)	MZ326000*	MZ403798*	MZ343047*	MZ403808*	MZ403794*
*Hedyotisovata* Thunb. ex Maxim.	China: Guobin Jiang et al. 1508 (IBSC)	MZ326003*	MZ403799*	MZ343053*	MZ403807*	MZ403793*
*Hedyotisshenzhenensis* Tao Chen	China: Ruijiang Wang et al. 1262-1 (IBSC)	JF976502	JF700101	JX111276	JF699805	JX111350
*Hedyotisuncinella* Hook. & Arn.	China: Ruijiang Wang 1217 (IBSC)	JF699963	JF700113	JX111282	JF699814	JX111356
*Involucrellachereevensis* (Pierre ex Pit.) Neupane & N. Wikstr.	Thailand: Suphuntee 799 (ODU)	KP994258	KR005743	KR005803	/	/
*Involucrellacoronaria* (Kurz) Neupane & N. Wikstr.	China: Xing Guo & Ping Yang 22-1 (IBSC)	JX111218	JX111104	JX111270	JX111177	JX111344
*Leptopetalumbiflorum* (L.) Neupane & N. Wikstr.	Singapore: Ruijiang Wang SIN03 (IBSC)	JX111238	JX111120	JX111302	JX111192	JX111376
*Leptopetalumpteritum* (Blume) Neupane & N. Wikstr.	China: Ruijiang Wang 1478 (IBSC)	JF699944	JF700094	/	JF699799	/
Oldenlandia capensis L. f. var. capensis	Zambia: Dessein et al. 843 (BR)	AM939496	EU557737	EU543048	/	EU543133
Oldenlandia corymbosa L. var. corymbosa	Singapore: Ruijiang Wang SIN02 (IBSC)	JX111239	JX111121	JX111306	JX111194	JX111380
*Oldenlandiaduemmeri* S. Moore	Uganda: W. H. Lewis 6018 (GH)	HE657744	HE657629	HE649881	/	/
*Oldenlandiaumbellata* L.	Sri Lanka: F. Fagerlind 3320 (S)	HE657674	HE657569	HE649806	/	/
*Oldenlandiawiedemannii* K.Schum.	Kenya: Luke & Luke 8362 (UPS)	AM939525	EU557756	EU543063	/	EU543151
*Parainvolucrellascabra* (Wall. ex Kurz) M.D. Yuan & R.J. Wang	China: Mingdeng Yuan & Yida Xu YS398_1 (IBSC)	MZ326006*	MZ403801*	MZ343069*	MZ403806*	MZ403796*
*Parainvolucrellascabra* (Wall. ex Kurz) M.D. Yuan & R.J. Wang	China: Mingdeng Yuan & Yida Xu YS398_2 (IBSC)	MZ326007*	MZ403802*	MZ343070*	MZ403805*	MZ403797*
*Parainvolucrellascabra* (Wall. ex Kurz) M.D. Yuan & R.J. Wang	China: Mingdeng Yuan & Yida Xu YS399 (IBSC)	MZ326008*	MZ403803*	MZ343071*	MZ403804*	MZ403795*
*Parainvolucrellascabra* (Wall. ex Kurz) M.D. Yuan & R.J. Wang	Thailand: Neupane 183 (ODU)	KP994264	KR005751	KR005812	/	/
*Pentodonpentandrus* Vatke	Zambia: Dessein et al. 598 (BR)	AM939528	EU557759	EU543066	/	EU543154
*Scleromitrionangustifolium* (Cham. & Schltdl.) Benth.	China: Xing Guo & Ping Yang 12 (IBSC)	JF976506	JF700108	JX111297	JF699810	JX111370
*Scleromitriondiffusum* (Willd.) R.J. Wang	China: Xing Guo 51 (IBSC)	JF699932	JF700081	JX111308	JF699789	JX111381
*Scleromitrionkoanum* (R.J.Wang) R.J. Wang	China: Ruijiang Wang et al. 978 (IBSC)	JX111215	JX111101	JX111267	JX111174	JX111341
*Scleromitrionpinifolium* (Wall. ex G.Don) R.J. Wang	China: Ruijiang Wang 1231 (IBSC)	JX111240	JF700094	JX111311	JX111196	JX111384

Notes: “*” indicates the newly-seqenced fragments, “/” indicates the missing data.

## Results

### Phylogenetic analysis

**Figure 1.**
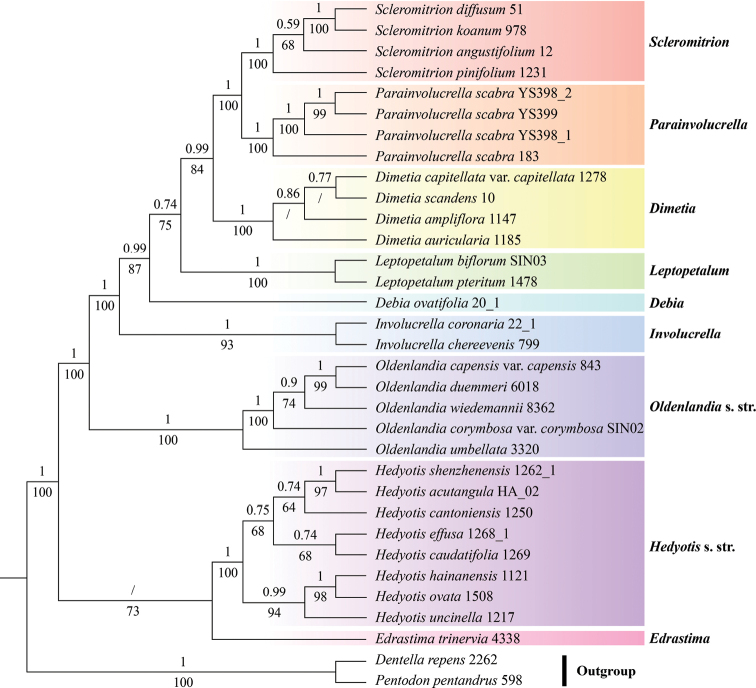
Phylogenetic relationships of the *Hedyotis-Oldenlandia* complex derived from a combined analysis of ITS and plastid *pet*D, *rps*16, *trn*H-*psb*A and *trn*L-F. Bayesian Posterior Probability (PP ≥ 0.5) and Bootstrap values (BS ≥ 50%) are indicated above and below the branches, respectively.

The phylogenetic analysis, based on nuclear ITS and four chloroplast DNA regions (*pet*D, *rps*16, *trn*H-*psb*A and *trn*L-F), generated an almost identical tree to that of Neupane et al. (2015). It showed that all the samples of *Hedyotisscabra* cluster into an independent clade which is sister to *Scleromitrion* with robust support (PP = 1, BS = 100, Fig. 1). In addition, the morphological similar species, *Involucrellacoronaria*, nested in the *Involucrella* clade (PP = 1, BS = 93, Fig. 1) and is sister to the lineage of (*Debia* clade + (*Leptopetalum* clade + (*Dimetia* clade + (*Scleromitrion* clade + *H.scabra* clade)))) with robust support (PP = 1, BS = 100, Fig. 1).

### Taxonomic treatment

Based on the morphological and palynological differences between *Hedyotisscabra* and *Scleromitrion*, as well as the molecular evidence, a new genus is proposed here.

#### Parainvolucrella

Taxon classificationPlantae

R.J. Wang gen. nov.

9F9A3C7F-1391-5B10-8600-0D26F5892544

urn:lsid:ipni.org:names:77218849-1

##### Note.

Annual or perennial herbs. Stem decumbent. Inflorescences terminal, congested-cymose, involucrated. Flowers heterostylous; petals 4; ovary 2-loculed, ovules many. Pollen 3-colporate; tectum double microreticulate. Fruits indehiscent. Seeds trigonous; testa reticulate.

##### Type.

*Parainvolucrellascabra* (Wall. ex Kurz) M.D. Yuan & R.J. Wang (*Hedyotisscabra* Wall. ex Kurz)

#### Parainvolucrella scabra

Taxon classificationPlantae

(Wall. ex Kurz) M.D. Yuan & R.J. Wang comb. nov.

D61387EF-8E87-551E-9505-38066BD9FB03

urn:lsid:ipni.org:names: 77218850-1

Figs 2 , 3

Basionym: Hedyotisscabra Wall. ex Kurz, J. Asiat. Soc. Bengal, Pt. 2, Nat. Hist. 46(2): 133, 136 (1877). Type: MYANMAR. from Martaban down to Upper Tenasserim, *Wall. Cat. 880* (holotype: CAL; isotypes: G [G00436284!; G00436285!]; K [K001110148!; K001110149! K000031881!]).
Synonym: Scleromitrionscabrum (Wall. ex Kurz) Neupane & N.Wikstr., Taxon 64(2): 317 (2015)

##### Description.

Annual or perennial herbs. Stems decumbent, ca. 1 m long, roughly angular, usually rooted at nodes; branches ascending to 30 cm high. Leaves opposite, subsessile to petiolate, petiole to 3 mm long; blades 2.0–7.0 × 1.0–3.0 cm, narrowly ovate to ovate, apex acute, base cuneate; leaf scabrid adaxially and along the veins abaxially; mid-rib depressed adaxially and prominent abaxially; secondary veins 5–6 on each side. Stipules ca. 3.0 × 2.0 mm, triangular, fimbriate with tipped colleters, excurved, pubescent abaxially. Inflorescence terminal, (2–)3–8(–12)-flowered, congested-cymose, usually subtended by 4 involucral leaves; peduncle subsessile; bracts 2–3 mm long, narrowly ovate, scabrid; bracteoles ca. 1 mm long, truncate to broadly ovate-triangular, fimbriate with tipped colleters, glabrous. Flowers heterostylous, pedicels to 0.8 mm long. Hypanthium ca. 0.8 mm long, obconic, 4 longitudinal projections against the lobes; lobes 4, ca. 1.5 × 0.4 mm long, narrowly triangular to narrowly oblong, scabrid. Corolla white, tube 1.5–2.0 mm long, glabrous abaxially and pubescent adaxially; lobes 4, 2.3–2.8 × 0.7–0.8 mm, oblong. Stamens 4, anthers 0.6–0.7 mm long. Stigma bilobed, 0.5–0.6 mm long, papillate. Longistylous flowers: stamens included, filaments adnate to the base of corolla tube, filaments ca. 2 mm long; styles ca. 4.3 mm long, exserted, included part pubescent, stigma ellipsoid. Brevistylous flowers: stamens included; filaments adnate to the base of corolla tube, filaments ca. 5.6 mm long; styles ca. 2 mm long, exserted, pubescent, stigma clavate. Fruits ca. 2.1 × 2.3 mm, subglobose, with 4 longitudinal projections when young, scabrid, indehiscent. Seeds trigonous, 0.4–0.5 mm, numerous, black; testa reticulate.

**Figure 2.**
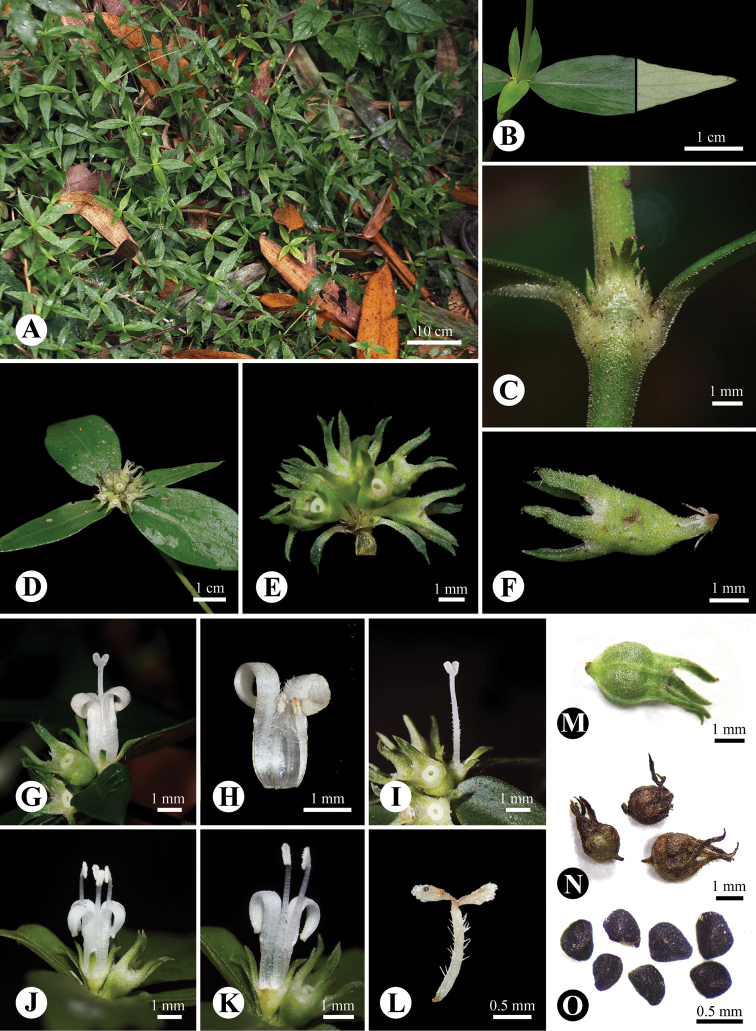
*Parainvolucrellascabra* (Wall. ex Kurz) M.D. Yuan & R.J. Wang **A** habit **B** leaf adaxial (left) and abaxial (right) surface **C** stem and stipule **D** infructescence with four involucral leaves **E** infructescence with bracts **F** calyx with bracteole at base **G–I** longistylous flower **J–L** brevistylous flower **M, N** fruits **O** seeds.

**Figure 3.**
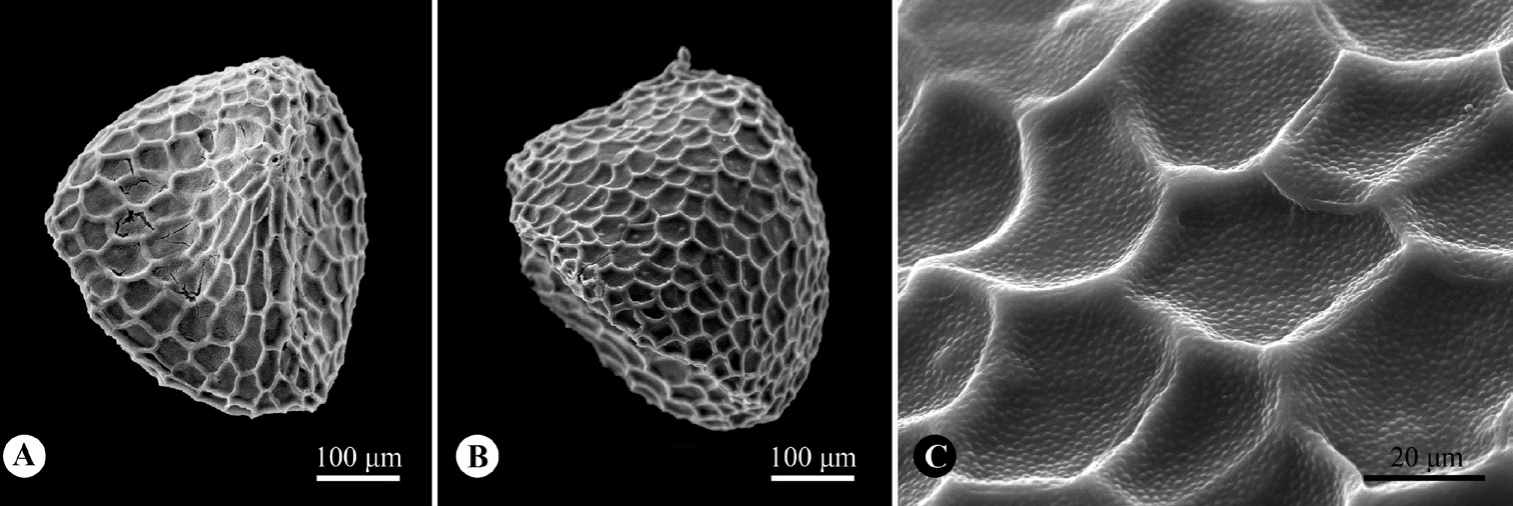
Seed morphology of *Parainvolucrellascabra***A** ventral side **B** dorsal side **C** testa ornamentation.

##### Phenology.

Flowering from July to September; fruiting from October to December.

##### Etymology.

The generic name *Parainvolucrella* alludes to similarity to *Involucrellacoronaria* in possessing terminal inflorescence subtended by four involucral leaves.

##### Distribution and habitat.

Bangladesh, India, Myanmar, Thailand and Vietnam (Fukuoka 1970; Dutta and Deb 2004), and China (new record). Only one subpopulation including about 200 individuals was found in dense bamboo forest and at the edge of the forest nearby the Nonggang National Nature Reserve. The habitat there belongs to a tropical monsoon climate, main associated species are *Dendrocalamuslatiflorus* Munro (Poaceae) and *Centothecalappacea* (L.) Desv. (Poaceae).

##### Palynology.

**Figure 4.**
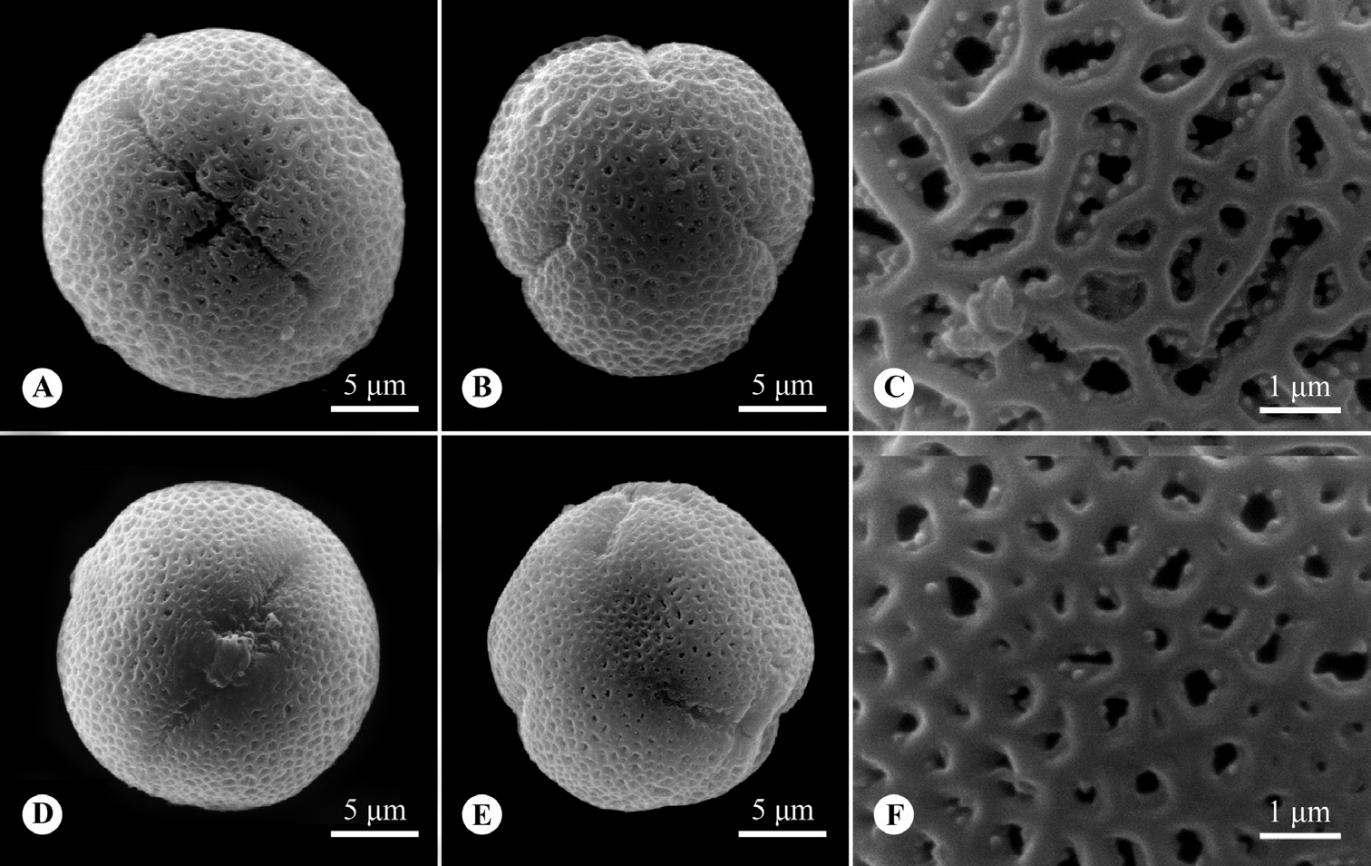
Pollen morphology of *Parainvolucrellascabra* (**A–C** from Mingdeng Yuan & Yida Xu YS398, brevistylous flower **D–F** from Mingdeng Yuan & Yida Xu YS399, longistylous flower) **A, D** equatorial view **B, E** polar view **C, F** double microreticulate ornamentation of mesocolpium.

Monads, isopolar and prolate-spheroidal, with 3-colporate apertures; the tectum is double microreticulate, with a psilate suprareticulum and a microechinate infrareticulum. The pollen size is 22.2 (20.9–23.7) × 20.2 (18.3–21.8) μm with P/E value 1.10 in brevistylous flowers (Fig. 4A–C); and 20.2 (18.5–21.2) × 19.0 (16.6–20.6) μm with P/E value 1.06 in longistylous flowers (Fig. 4D–F).

##### Additional specimens examined.

**China.** Guangxi Zhuang Autonomous Region: Chongzuo City, Longzhou County, Zhubu Town, Nonggang Village, 1 Nov 1978, Nonggang Investigation Team 11263 (IBK!); same locality, 22°29'16"N, 106°56'13"E, elev. 287 m, 29 Oct 2020, Ming-Deng Yuan & Yi-Da Xu YS398, YS399 (IBSC!); same locality, 22°29'22"N, 106°56'11"E, elev. 290 m, 2 Feb 2021, Ming-Deng Yuan YS407 (IBSC!); Zhubu Town, Lenglei Village, 9 Oct 1979, Nonggang Investigation Team 20457 (GXMI!). **India.** India orientalis: in Bengalia circa Calcuttam, J.W.Helfer 40 (P03904580). **Thailand.** Kampeng: A.F.G. Kerr 6161 (SING!); Tak: Ban Musoe, 22 Jul 1973, Gen Murata et al. 16719 (P03904581).

### Key to the genera of the *Hedyotis-Oldenlandia* complex in China

**Table:** 

1	Decumbent or prostrate herbs or climbers	**2**
–	Erect or ascending herbs, subshrubs or shrubs	**5**
2	Herbs; venation triplinerved inconspicuously above base; flowers homostylous	*** Edrastima ***
–	Herbs or climbers; pinnated venation; flowers heterostylous	**3**
3	Climbers	*** Dimetia ***
–	Decumbent or prostrate herbs	**4**
4	Stipules triangular, fimbriate with tipped colleters; inflorescence terminal, subtended by four leaves	*** Parainvolucrella ***
–	Stipules broadly triangular, apex spinous; inflorescence terminal or axillary, without subtended leaves	*** Dimetia ***
5	Shrubs or subshrubs	*** Hedyotis ***
–	Herbs	**6**
6	Inflorescence terminal, subtended by two or four leaves	**7**
–	Inflorescence terminal or axillary, without subtended leaves	**8**
7	Inflorescence large and loose, peduncles and pedicels long	*** Debia ***
–	Inflorescence small and congested, peduncles and pedicels subsessile	*** Involucrella ***
8	Fruits winged conspicuously or inconspicuously	*** Leptopetalum ***
–	Fruits wingless	**9**
9	Herbs gracile; growing in limestone area	*** Involucrella ***
–	Herbs robust; growing in non-limestone area	**10**
10	Stipules papery, hard, entire or fimbriate; flower homo- or heterostylous; fruits dehisce diplophragmously	*** Hedyotis ***
–	Stipules membrane, fimbriate; flower homostylous; fruits dehisce loculicidally	**11**
11	Stamens and stigma included in corolla tube	*** Oldenlandia ***
–	Stamens and styles exserted from corolla tube	*** Scleromitrion ***

## Discussion

The plant habit, stipule shape, inflorescence position, flower distyly and the dehiscent pattern of the fruits are of diagnostic significance in the different genera of the *Hedyotis-Oldenlandia* complex (Dutta and Deb 2004). Several successive field collections observed that the fruits of *Hedyotisscabra* are completely indehiscent, which was obscurely diagnosed by Hooker (1880) and incorrectly described by Dutta and Deb (2004). *Hedyotisscabra* differs from *Scleromitrion* by the terminal inflorescences with involucral leaves (vs. axillary or terminal and axillary in the uppermost leaf axils in *Scleromitrion*), the heterostylous flowers (vs. homostylous in *Scleromitrion*), pollen grains tectum double microreticulate, with psilate suprareticulum and microechinate infrareticulum (vs. rugulate tectum with microechinate muri in *Scleromitrion*) and indehiscent fruits (vs. loculicidally dehiscent in *Scleromitrion*). On the other hand, *Parainvolucrellascabra* is similar to *Involucrellacoronaria* with respect to their terminal inflorescence subtended by involucral leaves, heterostylous flowers and indehiscent fruits, but *Parainvolucrella* has decumbent habit (vs. erect or ascending in *Involucrellacoronaria*), young fruits with 4 longitudinal projections (vs. smooth surfaces in *Involucrellacoronaria*) and trigonous seeds with no pits on the surface (vs. ellipsoidal and 3–5 pitted seeds in *Involucrellacoronaria*) (Table 2).

**Table 2.** Morphological comparison of the *Hedyotis*-*Oldenlandia* complex distributed in China.

Taxon	Habit	Stipules	Flowers	Fruits	Seeds	Pollen
***Debia*** Neupane & N. Wikstr.	Annual small herbs, erect	Papery, broadly triangular, fimbriate with tipped colleters	Homostylous with exserted stigma and stamens	Compressed globose, loculicidally dehiscent	Conoidal with deeply depressed exotesta, anticlinal boundaries nearly straight or rounded	3-colporate, perforate tectum with psilate muri
***Dimetia*** (Wight & Arn.) Meisn.	Perennial herbs or subshrubs, prostrate, decumbent or climber	Papery, truncate, broadly rounded or broadly triangular, spinous	Heterostylous	Subglobose to ellipsoidal, dehiscent diplophragmously or indehiscent	Dorsiventrally flattened or trigonous, reticulate, anticlinal boundaries nearly straight	3- or 4-colporate, double microreticulate tectum with psilate suprareticulum and microechinate infrareticulum
***Edrastima*** Raf.	Annual small herbs, decumbent	Membranous, truncate, fimbriate with tipped colleters	Homostylous with exserted stigma and stamens	Subglobose, loculicidally dehiscent	Trigonous to ellipsoidal, reticulate, anticlinal boundaries nearly straight	3-colporate, microreticulate tectum with psilate muri
***Hedyotis*** L.	Perennial herbs to shrubs, erect or ascending	Papery, triangular, entire to fimbriate with tipped colleters	Heterostylous or rarely homostylous with exserted stigma and stamens	Ellipsoidal, dehiscent diplophragmously or rarely indehiscent	Dorsiventrally flattened, reticulate, anticlinal boundaries nearly straight	3- or 4-colporate, double microreticulate tectum with psilate suprareticulum and microechinate infrareticulum
***Involucrella*** (Hook. f.) Neupane & N. Wikstr.	Annual herbs, erect or ascending	Papery, triangular or truncate, margin fimbriate or acicular spinous with tipped colleters	Heterostylous or rarely homostylous with included stigma and stamens	Hemispherical to ellipsoidal, loculicidally dehiscent or indehiscent	Ellipsoidal, 3–5 pitted, anticlinal boundaries nearly straight or undulate	3- or 4-colporate, double microreticulate tectum with psilate suprareticulum and microechinate infrareticulum
***Oldenlandia*** L.	Annual small herbs, erect or ascending	Membranous, flabellate or broadly rounded, fimbriate with tipped colleters	Homostylous with included stigma and stamens	Globose to ellipsoidal, loculicidally dehiscent	Trigonous, reticulate, anticlinal boundaries nearly straight	3- or 4-colporate, microreticulate tectum with psilate muri
***Parainvolucrella*** R.J. Wang	Annual or perennial herbs, decumbent	Papery, triangular, fimbriate with tipped colleters	Heterostylous	Subglobose, 4 longitudinal projections when young, indehiscent	Trigonous, reticulate, anticlinal boundaries nearly straight	3-colporate, double microreticulate tectum with psilate suprareticulum and microechinate infrareticulum
***Scleromitrion*** (Wight & Arn.) Meisn.	Annual small herbs, erect or ascending	Membranous, triangular to rounded, fimbriate with tipped colleters	Homostylous with exserted stigma and stamens	Subglobose, loculicidally dehiscent	Trigonous to conoidal, reticulate, anticlinal boundaries nearly straight	3- or 4-colporate, rugulate tectum with microechinate muri
***Leptopetalum*** Hook. & Arn.	Annual small herbs, erect	Papery, triangular or broadly triangular, fimbriate with tipped colleters	Homostylous with included stigma and stamens	Obconical, winged, loculicidally dehiscent	Ellipsoidal with deeply depressed exotesta, anticlinal boundaries undulate	3-colporate, microreticulate tectum with psilate muri

Based on the combined nuclear (ITS, ETS) and plastid (*pet*D, *rps*16) data, Neupane et al. (2015) did not provide a well-resolved phylogenetic tree to support the placement of *Hedyotisscabra* as sister to the remainder of *Scleromitrion* in the *Hedyotis-Oldenlandia* complex, neither did Gibbons (2020). In addition, it seemed that the morphological confliction between the *H.scabra* and *Scleromitrion* and the phylogenetic exclusion of *H.scabra* from *Scleromitrion* clade were overlooked before making the new combination by Neupane et al. (2015). Our further integrated analysis, based on the morphological incongruence and the robust phylogenetic support (BS = 100, PP = 1), based on nrITS and plastid *pet*D, *rps*16, *trn*H-*psb*A and *trn*L-F, elucidated the taxonomic and phylogenetic confusions and thus the new monotypic genus *Parainvolucrella* is proposed here.

## Supplementary Material

XML Treatment for Parainvolucrella

XML Treatment for Parainvolucrella scabra
